# Producing knowledge by admitting ignorance: Enhancing data quality through an “I don’t know” option in citizen science

**DOI:** 10.1371/journal.pone.0211907

**Published:** 2019-02-27

**Authors:** Marina Torre, Shinnosuke Nakayama, Tyrone J. Tolbert, Maurizio Porfiri

**Affiliations:** 1 Department of Mechanical and Aerospace Engineering, New York University Tandon School of Engineering, Brooklyn, New York, United States of America; 2 Department of Biomedical Engineering, New York University Tandon School of Engineering, Brooklyn, New York, United States of America; University of Ulm, GERMANY

## Abstract

The “noisy labeler problem” in crowdsourced data has attracted great attention in recent years, with important ramifications in citizen science, where non-experts must produce high-quality data. Particularly relevant to citizen science is dynamic task allocation, in which the level of agreement among labelers can be progressively updated through the information-theoretic notion of entropy. Under dynamic task allocation, we hypothesized that providing volunteers with an “I don’t know” option would contribute to enhancing data quality, by introducing further, useful information about the level of agreement among volunteers. We investigated the influence of an “I don’t know” option on the data quality in a citizen science project that entailed classifying the image of a highly polluted canal into “threat” or “no threat” to the environment. Our results show that an “I don’t know” option can enhance accuracy, compared to the case without the option; such an improvement mostly affects the true negative rather than the true positive rate. In an information-theoretic sense, these seemingly meaningless blank votes constitute a meaningful piece of information to help enhance accuracy of data in citizen science.

## Introduction

Participation of non-trained people in scientific research projects, often called “citizen science”, has been continuously gaining popularity [1–4]. Since the first massive citizen participation in bird counting in 1900 [5,6], the number of projects has considerably increased, covering many research disciplines, from ecology [7] to biology [8], astronomy [9], and geography [10,11]. Popularity of citizen science has further expanded with the accessibility to computers and mobile devices [6,12–14]. Through online platforms, volunteers can remotely contribute to various disciplines by performing tasks such as classifying galaxies [15,16], DNA sequences alignment [17], analyzing and modeling protein structures [18], and identifying cancer cells [19]. However, one of the major challenges in citizen science is guaranteeing a satisfactory level of data quality, considering that most of the participants are not professionally trained in the specific field of research [20–22].

A powerful method to deal with the so-called “noisy labeler problem” is the estimation-maximization algorithm [23]. Using the data on labelers’ responses on multiple tasks, the algorithm infers posterior distributions of correct answers and labelers’ error rates through maximum likelihood estimation [23]. The algorithm has been extended to include the estimation of task difficulties [24,25] and the possibility of correcting labelers’ biases [26], toward improved prediction of correct answers. However, these methods often require a large sampling pool to attain high accuracy [27], and, therefore, are not practical for several citizen science projects where the number and effort of volunteers are limited. Further, these methods are designed for static data, which demand redundancy in labeling efforts when the task difficulty is not known in advance. Considering that volunteers’ effort is a valuable and constrained resource for the researchers, an economical solution would be to re-direct the participants to tasks that would benefit from more responses.

Dealing with the problem of limited effort by participants in citizen science is similar to optimal task allocation among crowdsourcing workers under a limited budget, where practitioners aim to reduce the total cost while maintaining a desired accuracy. Intensive research has been focused on the design of algorithms that dynamically allocate instances when crowdsourcing workers sequentially enter the system [28–33]. Agreement on each instance is quantified through the information-theoretic notion of entropy. Entropy is a measure of the uncertainty of a random variable, where high entropy relates to a highly stochastic state, and low entropy represents a predictable, nearly deterministic one [34]. In the context of labeling, the entropy of a specific instance measures the level of agreement among labelers, which is related to the accuracy of the responses when the labels are aggregated [35,36]. Based on entropy and its derivative metrics, the framework of sequential task allocation attempts to dynamically select instances that maximize a utility function under a Markov decision process [28,29,33].

Dynamic task allocation presumes that workers label each instance without the possibility to avoid labeling and report an answer like “I don’t know”. In the estimation-maximization algorithm, it is necessary that labelers select a response, rather than choosing a hypothetical “I don’t know” option, whereby knowledge about a wrong selection is useful information for estimating individual error rates. Just as dynamic task allocation in crowdsourcing projects has stayed away from an “I don't know” option, so did citizen science, although for a different reason. In citizen science, an “I don’t know” option has been proposed to be detrimental, because it might reduce the output of volunteers who could overuse it [37]. However, it is presently unknown whether the same rationale applies to dynamic task allocation that involves a fewer number of volunteers per instance. In this situation, an “I don’t know” option might increase accuracy by providing further information about the confidence of the aggregated responses when entropy is used to determine the level of agreement among volunteers. For example, volunteers might frequently choose an “I don’t know” option when an image is difficult to classify, whereas they might select correct labels when an image is simple to classify. Thus, an “I don’t know” option could provide additional information about the difficulty of the task, but research to address this hypothesis is presently lacking.

Toward illuminating the influence of an “I don’t know” option on data quality within entropy-based dynamic task allocation, we conducted a citizen science project in which volunteers performed binary classification tasks with an “I don’t know” option. The study was carried out within the Brooklyn Atlantis Project [38], which entails monitoring the environment of the Gowanus Canal (Brooklyn, NY), a highly polluted body of water in the U.S. Volunteers were presented with images of the Canal and asked to classify the objects in the images, by assessing whether they could pose a threat to the environment. Using this dataset, we apply the notion of entropy to measure the level of agreement among volunteers with respect to their responses in a specific image. Entropy is computed in three different ways, which contrast in how the “I don’t know” is treated. Specifically, entropy is computed by (1) using only binary labels, (2) including “I don’t know” as a third class, and (3) randomly reassigning “I don’t know” into either label, mimicking the situation where volunteers are forced to choose one when they do not know. We adopt a simplified task allocation procedure where tasks are randomly allocated to volunteers until the entropy falls below a chosen threshold. The entropy of each task is progressively updated to determine whether the task should require more responses from additional volunteers. We compare accuracy as a measure of data quality across the cases in which “I don’t know” is treated differently.

## Methods

### Dynamic task allocation procedure

We used the information-theoretic notion of entropy [34] to determine whether an instance requires more labels from additional volunteers. Entropy (*H*) is a measure of uncertainty of a random variable, quantified as

$$H=-\sum_{i=1}^{n}p_{i}\log_{2}p_{i},$$

where *p*_*i*_ is the probability of observing the category *i* among *n* possible categories. When applied to an image classification task, images with high entropy indicate a large uncertainty in classification among volunteers, whereas those with zero entropy identify consensus among volunteers.

In our procedure, volunteers sequentially enter the system and classify images randomly taken from an image repository into pre-defined categories. As a new volunteer classifies the images, the entropy of each image is progressively updated. The system assesses whether the image requires further analysis by new volunteers, by comparing the current entropy of the image with a certain threshold. When the entropy lowers below the threshold, the image is deemed processed and removed from the repository, and no further labeling is conducted by new volunteers. If the entropy is above the threshold, then the image stays in the repository, subjected to further labeling by new volunteers. Although there exist more sophisticated algorithms to intelligently allocate items to classifiers based on the transient entropy and similar metrics [28,29,33], we chose random task allocation to focus on our research question, which is to illuminate the influence of an “I don’t know” option on data quality.

### Data collection

The experiment was framed in the context of a citizen science project for monitoring the environmental health of the Gowanus Canal (Brooklyn, NY, USA). To obtain information about the status of the environmental health of the canal, volunteers were asked to analyze the images of the canal and identify the presence of objects that could constitute a threat for the environment. The images were taken by the aquatic robot designed by our team as part of the Brooklyn Atlantis Project [38], which, over the years, was used by our group to address a number of important areas in citizen science, including face-to-face interactions between volunteers and researchers [39], the effect of individual curiosity on contribution [40], motivations [41–43], and the potential of integrating rehabilitation tasks into citizen science [44–47].

The robot is able to navigate on the water surface of the Canal and collect water quality data (pH, conductivity, salinity, temperature, and oxygen concentration) and images, through onboard sensors and a camera above the water surface. The images taken by the robot are uploaded on a temporary website built for this experiment, where volunteers can access them from their computers and mobile devices. The website was built using HTML and CSS for the design and JavaScript for functionalities such as sending data to the server. The web server was written in JavaScript using the Node.js runtime. The data are sent to and stored in a MySQL database, which is administrated using phpMyAdmin.

Before taking part in the project, participants were required to log in through either a Facebook profile or an email account. This login system allowed a one-time access with a personal account to guarantee that each participant performed the task only once. Upon accessing the website, participants were first presented with a short movie explaining the current pollution problems of the Canal and the objective of the project (S1 Video). To ensure that all participants received the same information, they were not allowed to take part in the project until the movie ended.

After the movie, participants proceeded to a practice session of image classification. The images contained objects (such as garbage, a bird, or a factory), which could give visual information of the environmental health of the Canal. In the practice session, participants were instructed to classify whether the object in the image would pose a threat to the water quality or wildlife by clicking either a “threat”, “no threat”, or “I don’t know” button below the image. Once the task was performed, the correct answer was displayed, along with a short description of the explanation.

**Fig 1:**
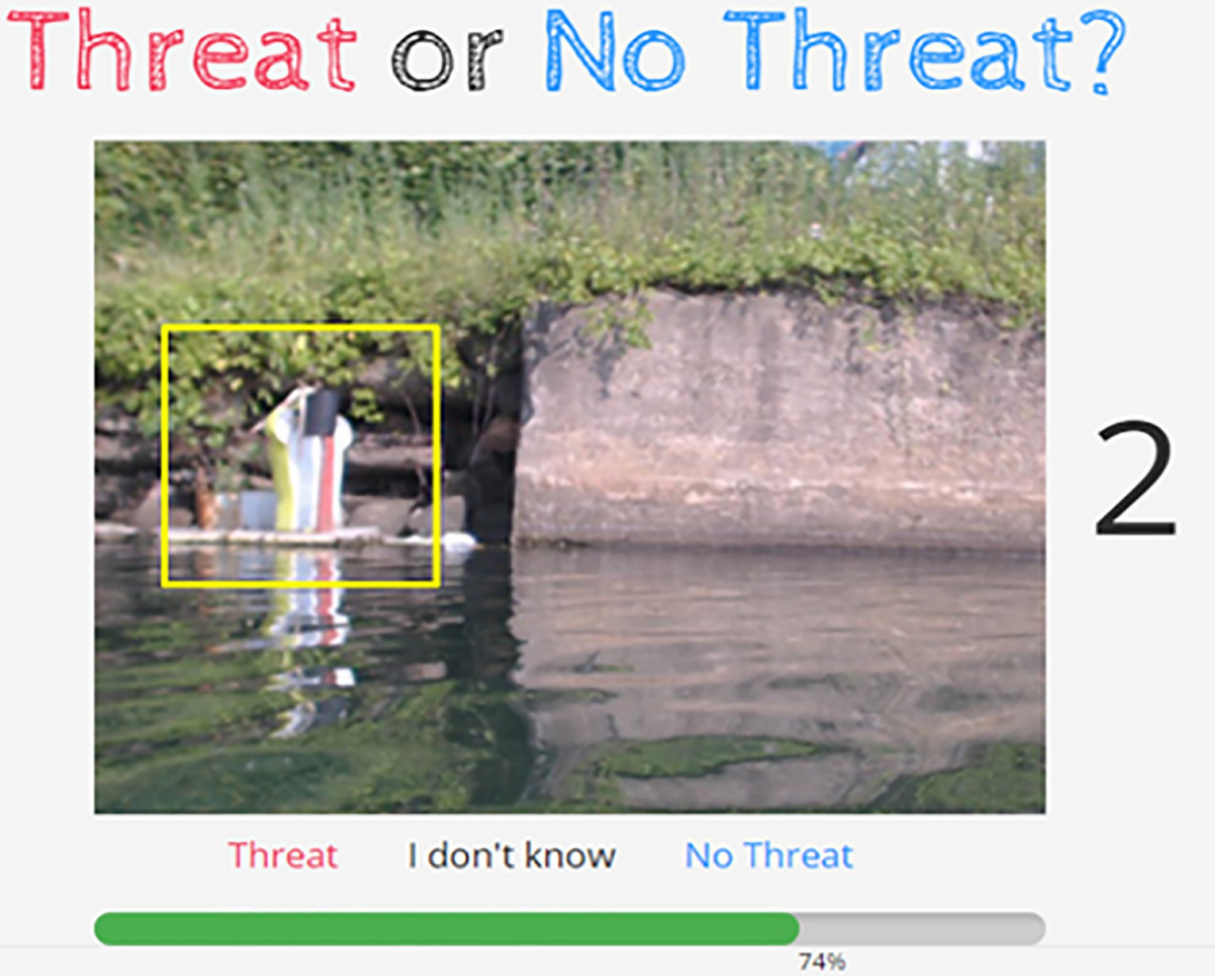
Screenshot of the image classification task. The object to be classified is highlighted by a rectangular frame. The number on the right (“2”) denotes the time remaining to answer the question in seconds. The bottom bar indicates the progress toward completing the classification of all images. The correct answer of this image is “no threat” (art installation).

Upon classifying two objects in the practice session, participants proceeded to the main task in which the screen displayed 31 images consecutively for 5 seconds each (Fig 1). The time limit was fixed to grant that all participants would have the same amount of time to classify an image. Participants were asked to classify the highlighted object in each image into “threat”, “no threat”, or “I don’t know”, but this time, the correct answer was not displayed. When the participant did not select any answer in 5 seconds, it was recorded as “no answer”. The images were displayed in a random order for each participant to eliminate the influence of the display order on performance. For each participant, we recorded the anonymous user identification number generated from the website and the selected answer for each image. When a participant changed her/his opinion by clicking a different button within 5 seconds, we recorded only the last selection.

Before the experiment, all authors identified the correct answer of each image through careful examination and discussion. For example, we classified garbage, a factory with discharged water, or an oil spill on the water surface as “threat” to the environment, whereas a bird or an anthropic object within the human control, such as an art installation or a buoy, as “no threat” to the environment. We only used images that received unanimous consent within our research team to ensure that each of them could be properly associated with the correct answer (S1 File).

The data collection was carried out between February and June 2017. Participants were recruited through social media of New York University and the Gowanus Canal Conservancy (a local community), and by distributing flyers to passers-by in the neighborhood of the Gowanus Canal. In total, 94 volunteers were recruited in the project. All participants were over 18 years old and anonymous. The data collection was approved by the institutional review board of New York University (IRB-FY2016-184).

### Application to the citizen science data

We investigated the influence of an “I don’t know” option on data quality by assessing the performance of the system using the data collected from volunteers in our citizen science project. Specifically, we compared three cases that encompass hypothetical simulations: (1) volunteers were provided with three classes (“threat”, “no threat”, and “I don’t know”) but only “threat” and “no threat” were used to compute entropy, (2) all classes were used to compute entropy, and (3) each “I don’t know” choice was randomly reassigned to either “threat” or “no threat” when computing entropy. The latter case was intended to simulate the typical citizen science setting, in which a participant does not have access to the “I don't know” option.

In all the cases, we started by selecting a volunteer from the data set in a random order and allocating five images randomly drawn from the image repository, which initially contained 31 images. Collection of labels on each image was updated each time a new volunteer labeled the image. In the third case where volunteers were not provided with the “I don’t know” option, we reassigned it to either “threat” or “no threat” with an equal probability. In this way, we mimicked the situation where volunteers randomly chose either “threat” or “no threat” when they did not know which to choose. The entropy on each image was normalized between 0 and 1 for all three cases by dividing it by log_2_*N*, where *N* is the number of classes (*N* = 2 for cases 1 and 3, and *N* = 3 for case 2). An image was deemed processed and removed from the repository when the entropy fell below a certain threshold and it received at least three labels of “threat” or “no threat” combined. The latter condition was imposed to avoid the situation in which a first few votes on an image could lead to zero entropy by chance, while attempting to minimize the number of votes to process an image based on entropy. The procedure was continued until we exhausted either volunteers or images in the repository.

We assessed the performance of the three cases by varying the normalized entropy threshold from 0 to 1, with an interval of 0.1. Entropy threshold 0 means that an image was labeled unanimously, and 1 means that an image was removed from the repository when it received three “threat” and “no threat” combined, regardless of the level of agreement among volunteers. To test the situation where a smaller number of volunteers was available, we randomly sampled volunteers from 10 to 90, with an interval of 10. We performed 1,000 simulations each using R 3.4.0 [48].

### Evaluation of the system performance

We compared the system performance as a function of the entropy threshold for the three cases. To assess the quality of the system output, we aggregated the collection of labels into a single label for each processed image using simple majority voting on “threat” and “no threat”, due to its interpretability and robustness [49]. The votes for “I don’t know” were not included in the majority voting because our objective was to classify the images into either “threat” or “no threat”. Then, we quantified the accuracy of the system as the proportion of the number of images correctly classified over the total number of processed images, by comparing the aggregated label with the correct answer for each processed image. The quantity of the system output was scored as the total number of images processed.

To further examine the system performance, we compared the true positive rate (sensitivity) and the true negative rate (specificity) as a function of the entropy threshold for the three cases. To that end, first we classified each label of “threat” as a true or false positive and “no threat” as a true or false negative, by comparing it with the correct answer. Then, we tallied each occurrence on all processed images and calculated the true positive rate as the proportion of true positives over the sum of true positives and false negatives, and the true negative rate as the proportion of true negatives over the sum of true negatives and false positives.

To identify when volunteers opted for “I don’t know”, we documented the correct answers of the images that received “I don’t know” from volunteers. We counted the numbers of “threat” and “no threat” on such instances, and the frequency was compared with the one when volunteers actually labeled “threat” and “no threat” on the images, using a $\chi_{1}^{2}$ test.

## Results

### Summary of the citizen science data

In total, 94 volunteers contributed to the classification of the 31 images consisting of 11 “threat” and 20 “no threat” images. On average, volunteers selected 45.9% of the images as “threat” and 29.9% as “no threat”. They opted for “I don’t know” in 10.6% of the images and did not answer 13.6% of the images.

Reflecting the variation in classification difficulty among the images, each image received 1.1–90.4% of the 94 votes as “threat”, 1.1–92.6% as “no threat”, and 0–35.1% as “I don’t know”. Of the images, 5.3–26.6% were left without any choice. Among the images that contained “threat” objects, 71.8% of the votes correctly identified them as threat, ranging from 47.9 to 90.4% among the images, whereas 13.7% of the votes incorrectly identified them as no threat, ranging from 1.1 to 40.4% among the images. By contrast, among the images that contained “no threat” objects, 38.7% of the votes correctly identified them as no threat, ranging from 8.5 to 92.6% among the images, whereas 31.6% of the votes incorrectly identified them as threat, ranging from 1.1 to 69.1% among the images.

### Influence of “I don’t know” under entropy-based task allocation

**Fig 2:**
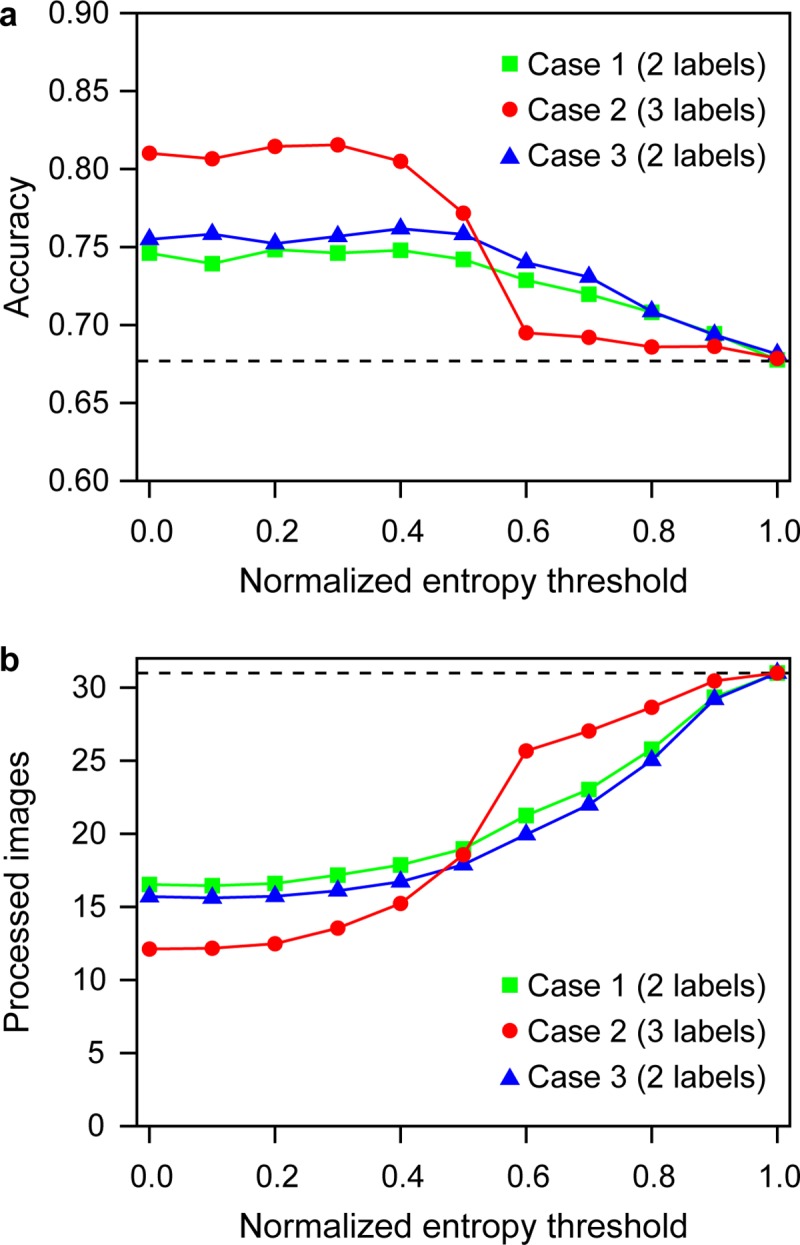
Performance of image classification as a function of the entropy threshold. (a) Accuracy and (b) number of image processed. Square: case 1, where image entropy is computed from two labels (“threat” and “no threat”), filled circle: case 2, where image entropy is computed from three labels (“threat”, “no threat”, and “I don't know”), triangle: case 3, where image entropy is computed from two labels (“threat” and “no threat”) after reassigning “I don’t know” to either class proportional to “threat” and “no threat” by all participants. Points and vertical lines are means and standard errors of 1,000 runs. Dotted lines correspond to the case, where no entropy threshold was applied (that is, the image is retired from the repository when it receives three labels of “threat” and “no threat” combined).

Sequential binary labeling with entropy-based task allocation increased data quality at the expense of data quantity, compared to the case in which no entropy threshold was implemented in task processing (Fig 2). In all the cases examined, a higher accuracy was attained with a smaller threshold, which corresponds to a higher level of agreement among volunteers. In case 2, where the “I don’t know” was used to compute entropy, the system was able to attain higher accuracy when the entropy threshold was below 0.5, compared to case 1, where the entropy was computed only with “threat” and “no threat”. However, the reverse trend was observed when the entropy threshold was above 0.5. By contrast, in case 3, where the “I don’t know” was randomly reassigned to either a “threat” or a “no threat” label in the entropy computation, the accuracy was virtually the same as in case 1, where only the original “threat” and “no threat” labels were used. Mirroring the improvement in accuracy, the number of images processed showed the opposite trend over entropy threshold. In addition, when “no answer” was included in “I don’t know”, or “no answer” was treated as an additional class, we observed the same trend as in case 2, where higher accuracy was attained at smaller entropy, compared to the cases where the entropy was computed only with “threat” and “no threat”.

**Fig 3:**
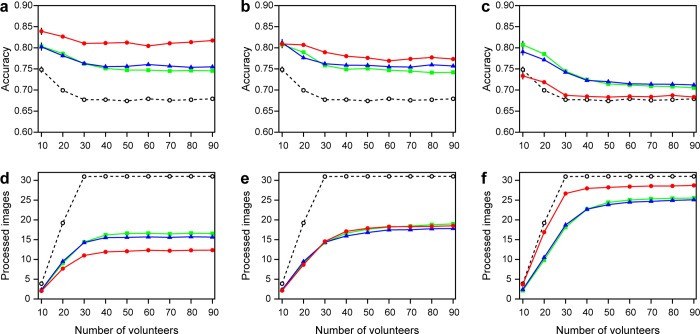
Performance of image classification over different numbers of volunteers. (a) Accuracy at entropy threshold 0.2, (b) at 0.5, and (c) at 0.8. (d) The number of processed images at entropy threshold 0.2, (e) at 0.5, and (f) at 0.8. Colors correspond to Fig 2 (square: case 1, filled circle: case 2, triangle: case 3, open circle: no entropy threshold).

The number of volunteers did not change the trend in accuracy (Fig 3). When a smaller number of volunteers performed image labeling, inclusion of an “I don’t know” option resulted in a higher accuracy with a smaller entropy threshold and in a lower accuracy with a larger entropy threshold. In all cases, accuracy increased when fewer volunteers were involved in image labeling.

**Fig 4:**
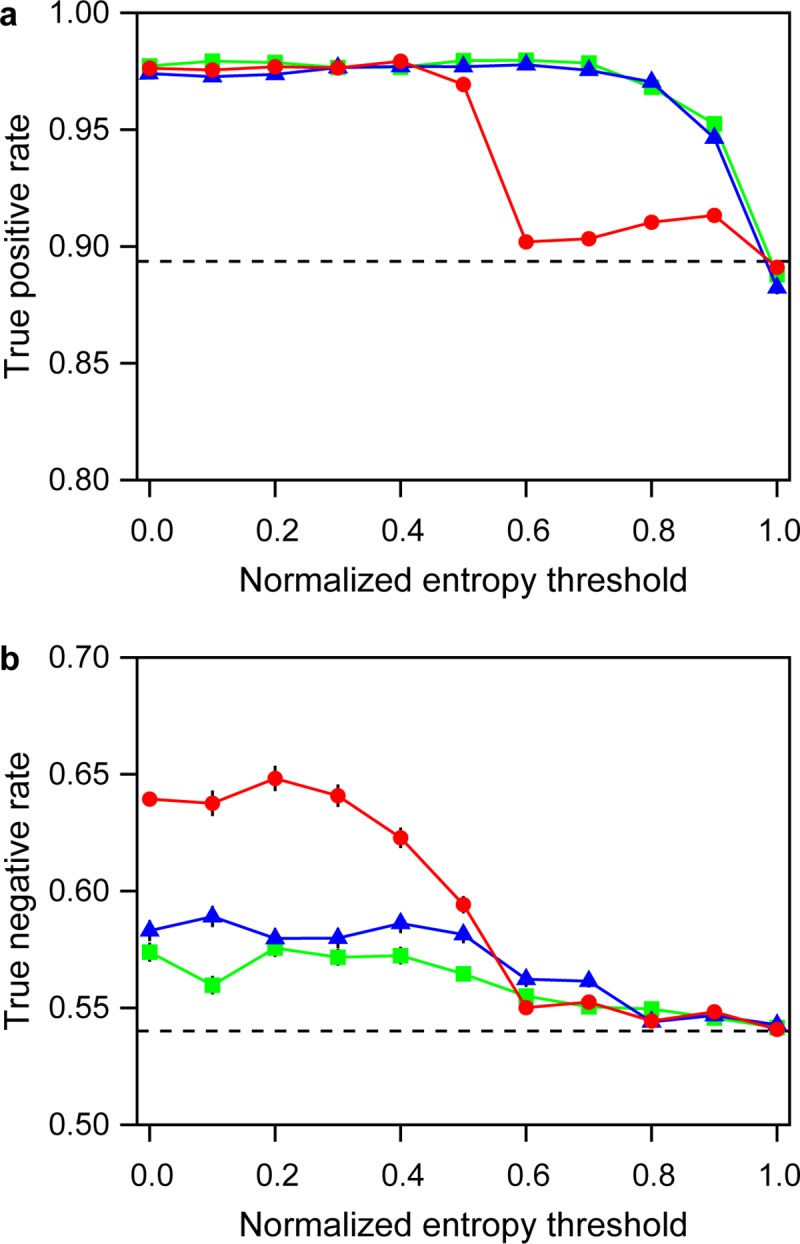
**(a) True positive rates and (b) true negative rates over entropy threshold.** Colors correspond to Fig 2 (square: case 1, filled circle: case 2, triangle: case 3).

The “I don’t know” option influenced the true positive rate and the true negative rate differently, as a function of the entropy threshold (Fig 4). When the entropy threshold was greater, the “I don’t know” option led to a lower true positive rate compared to the other cases in which the image entropy was computed using only two classes of “threat” and “no threat”. However, it achieved a similarly high true positive rate when the entropy threshold was below 0.5. By contrast, the “I don’t know” option led to greater improvement of the true negative rate with a decreasing entropy threshold, compared to the other two cases.

**Fig 5:**
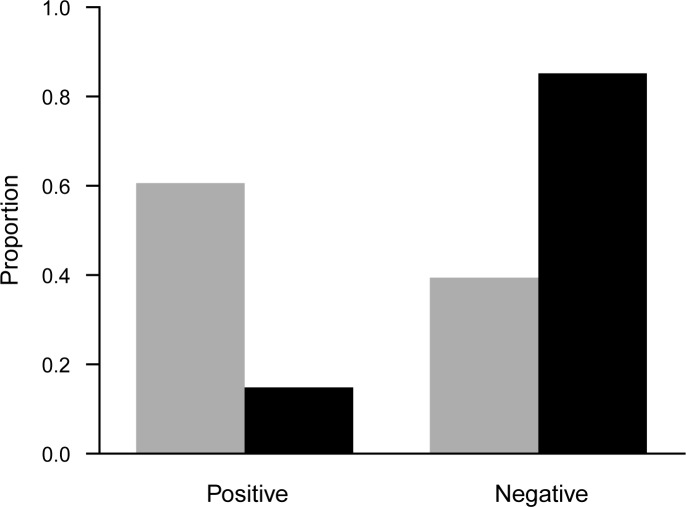
Proportion of the labels. Gray bars are observed proportions when participants labeled positive (threat) and negative (no threat). Black bars are the proportion of true answers when participants opted for “I don’t know”.

When volunteers labeled either “threat” or “no threat”, they were more likely to label “threat” over “no threat” (60.6% for “threat”), which significantly deviated from the distribution of the correct answers (35.5% for “threat”; $\chi_{1}^{2}$ = 7.02, *p* = 0.008). When they opted for “I don’t know”, the correct answer of those instances was significantly biased toward “no threat” (14.8% for “threat”; $\chi_{1}^{2}$ = 227.89, *p* < 0.001), compared to when they actually selected either “threat” or “no threat” (Fig 5).

## Discussion

In this study, we investigated the influence of an “I don’t know” option on data quality within a sequential task processing that utilizes the information-theoretic notion of entropy to dynamically allocate tasks among a limited number of volunteers. Confirming previous studies [28,29,33], we demonstrated that entropy is a useful tool to balance between accuracy of classification and the number of tasks completed. Without knowing the task difficulty or the volunteer reliability in advance, entropy can help improve classification performance, not at the expense of the workload of the volunteers. Within an entropy-based dynamic task allocation, our results show that providing volunteers with an “I don’t know” option is a useful means to further enhance accuracy. Compared to the case without such an option, the system was able to attain greater accuracy with the same number of volunteers. Thus, an “I don’t know” option allows for capitalizing on limited workload, by providing additional information that moderates accuracy of the classification, thereby offering an efficient and effective way to support data classification in citizen science.

The entropy of a task, scored based on volunteers’ responses, encapsulates information about the level of agreement among volunteers. In our citizen science project, images with high entropy indicate conflicting opinions among volunteers, leading to considerable uncertainty about the classification. On the other hand, images with low entropy indicate consensus among volunteers, suggesting clear classification of the images. By comparing volunteers’ responses with the correct answers, we found that when a lower entropy threshold is selected, the classification of the processed images is more accurate. The higher level of accuracy and the stronger agreement among participants reflect the difficulty of the images, confirming our intuition that entropy can be used as a proxy of task difficulty. In line with our observations, similar results were reported in the Snapshot Serengeti Project [37], where participants were asked to identify species through image classification. In that study, the correctly identified species through majority voting had lower standardized entropy, whereas incorrectly identified images had higher one [37]. Thus, the entropy of a task, scored based on participants’ responses, is a useful tool to determine whether the image requires further information from volunteers to be classified correctly, without knowing the true answer in advance. Entropy provides an indication of the reliability of the contributions, allowing researchers to selectively determine when data validation from experts is required [50]. Considering that the accuracy of entropy measures increases with the number of observations, it is possible to further improve the entropy-based task allocation by dynamically adjusting the entropy threshold proportional to the number of votes, such that entropy computed from a smaller number of votes would require a stricter threshold.

An “I don’t know” option affords volunteers with an opportunity to avoid random choice when they are not certain about the classification. Some citizen science platforms intentionally omit the possibility of these blank votes to avoid their overuse, and volunteers are forced to select one of the pre-defined classes to complete the task [37]. However, when entropy is applied to the image classification tasks, these blank votes that are seemingly not meaningful constitute a meaningful piece of information about the task. Specifically, when an image is difficult to classify, one would observe high entropy because of the large proportion of blank votes, in addition to splitting opinions between “threat” and “no threat” among volunteers. On the other hand, if the object in the image is simple to classify, volunteers may tend to answer correctly, thereby less likely cast blank votes. Additionally, the blank votes provide a beneficial piece of information about general knowledge of a specific question among citizen scientists. For example, questions with a high percentage of blank votes could offer a direction on which aspect should be emphasized in the training session in future citizen science projects.

Our results show that an “I don’t know” option moderates the tradeoff between the accuracy of the data analysis and the number of image processed. Compared to the hypothetical cases that do not use the “I don't know” option, the experimental configuration with such an option led to a higher accuracy with a smaller entropy threshold. At the same time, it led to a lower accuracy with a larger entropy threshold. The number of images processed mirrored the accuracy, with fewer images processed with a smaller entropy threshold. The same trends were observed when the analysis was conducted by fewer volunteers, demonstrating the generality of the result. The positive effect of an “I don’t know” option arises from the fact that it abates erroneous decision of the task by increasing the entropy through additional knowledge, thereby requiring stronger agreement among volunteers for the same entropy threshold. However, we observed the adverse effect of the “I don’t know” option on accuracy when the entropy thresholds were set high. This is because higher entropy thresholds are more likely to falsely detect agreement among volunteers on the task that received more “I don’t know” than “threat” or “no threat”. Such a false detection lead to lower accuracy by outweighing the positive effect brought about by the inclusion of the “I don’t know” answer. The adverse effect can easily be avoided by setting the entropy threshold smaller, or by simply adding an additional criterion to ensure that the entropy reflects the level of agreement between the labels of interest. Therefore, an “I don’t know” option can provide useful information toward enhancing data quality in citizen science projects when combined with entropy-based dynamic task allocation.

A multilabeling problem often ignores the asymmetry in the importance of labels, but researchers may want to place more emphasis on some labels over others, depending on their objectives. For example, spam email detection would be impractical with high false positive rates, whereas medical diagnostics would be dangerous with high false negative rates. Our results show that an “I don’t know” option can influence true positive rate and the true negative rate differently. Specifically, it led to greater improvement of the true negative rate compared to the true positive rate. This is because volunteers were more likely to opt for “I don’t know” when the correct answer was negative (“no threat”) than positive (“threat”). Consequently, the images received fewer erroneous negatives with the “I don’t know” option, thereby decreasing the false negative rate. Had we asked volunteers instead whether the objects in the images were beneficial to the environment, we should have observed a reverse result.

Although we demonstrated the benefit of an “I don’t know” option toward enhancing data quality, we cannot dismiss the possibility that forcing volunteers to choose binary answers could change their behavior. That is, if they did not have the “I don’t know” option, they might have exerted more effort to contribute to science, thereby influencing data quality. However, it is likely that accuracy would decrease further than a random choice, because the distribution of the observed labels submitted by volunteers was biased more toward “threat” than “no threat”, while the distribution of the true answers was the opposite. In such a case, it is possible to compensate the bias by applying a weight function during label classification if one knows the degree of bias in advance. Further research is required to understand how an “I don’t know” option would change motivations and effort in citizen science [51].

One of the most compelling challenges in citizen science projects is obtaining accurate information from citizens with no formal training. A common practice to guarantee an adequate accuracy involves the engagement of a large number of volunteers performing the same task and aggregate their answers [37,52]. In this study, we demonstrated that providing volunteers with an “I don’t know” option could enhance accuracy under entropy-based dynamic task allocation. The advantage could further be augmented by implementing more sophisticated task allocation algorithms [28,29,33]. The proposed framework does not require any assessment of volunteer reliability or task difficulty in advance, thereby laying the foundations for a powerful and efficient system that is easily customizable by researchers and applicable to different platforms.

## Supporting information

S1 Video Video clip explaining the current pollution problems of the Gowanus Canal and the objective of the project. (MP4)

S1 File Image used in this study. (ZIP)

S2 File Data collected and analyzed in this study. (TXT)
