# Supplementary figures and images for: Producing knowledge by admitting ignorance: Enhancing data quality through an “I don’t know” option in citizen science
Source: PLoS One. 2019 Feb 27;14(2):e0211907. doi: 10.1371/journal.pone.0211907 (PMC6392254; doi:10.1371/journal.pone.0211907)

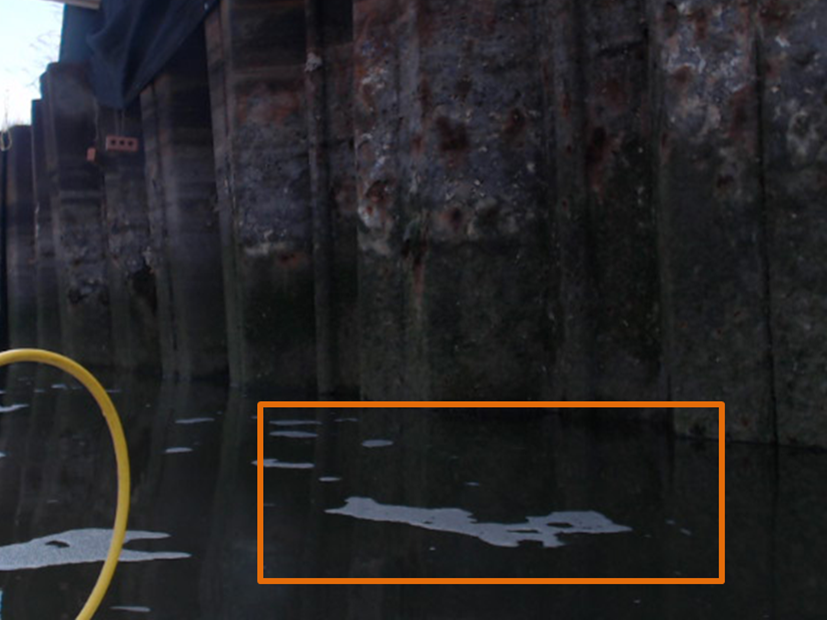

Supplement: S1 File — (ZIP) [file pone.0211907.s002.zip › Image14.png]

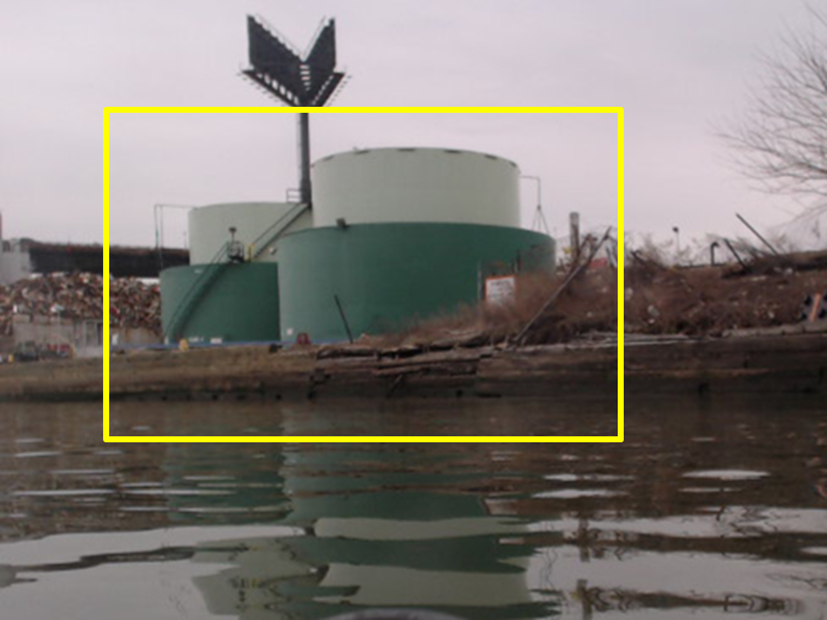

Supplement: S1 File — (ZIP) [file pone.0211907.s002.zip › Image28.png]

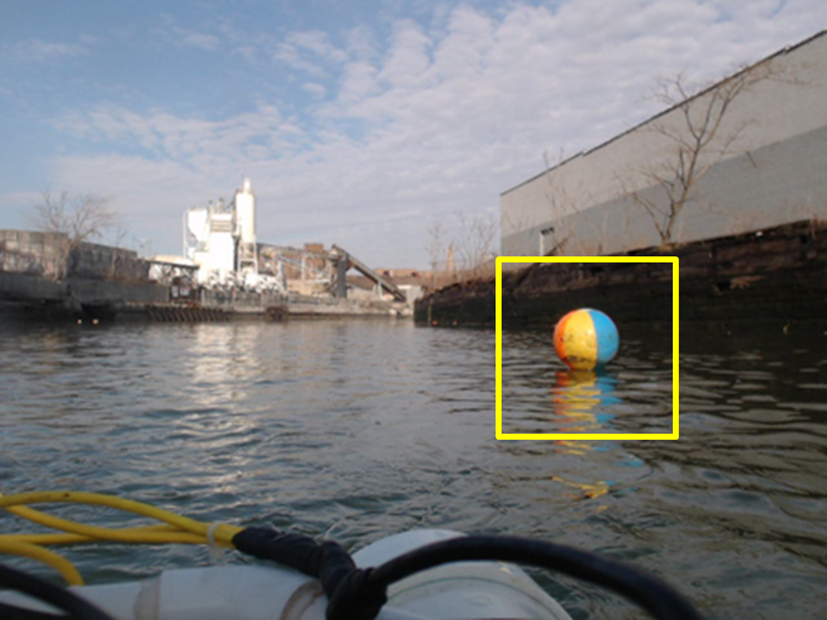

Supplement: S1 File — (ZIP) [file pone.0211907.s002.zip › Image29.png]

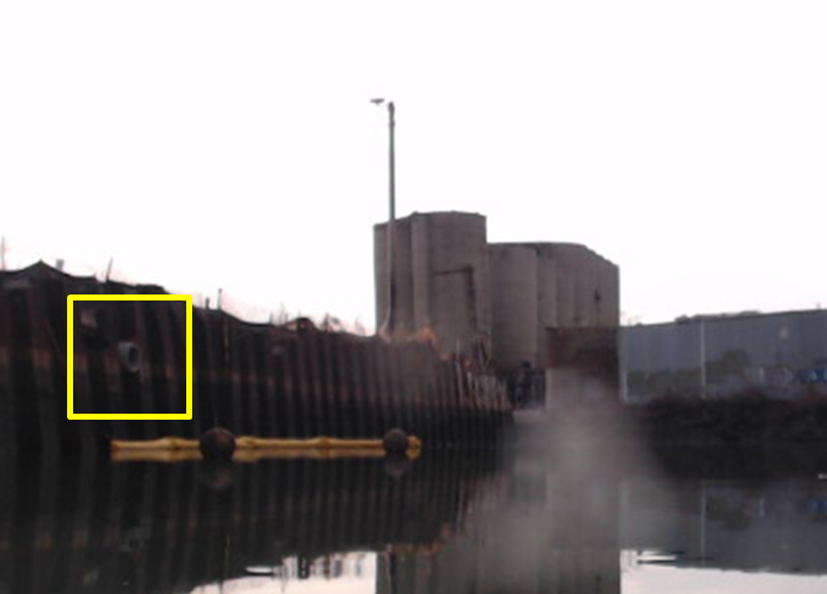

Supplement: S1 File — (ZIP) [file pone.0211907.s002.zip › Image15.png]

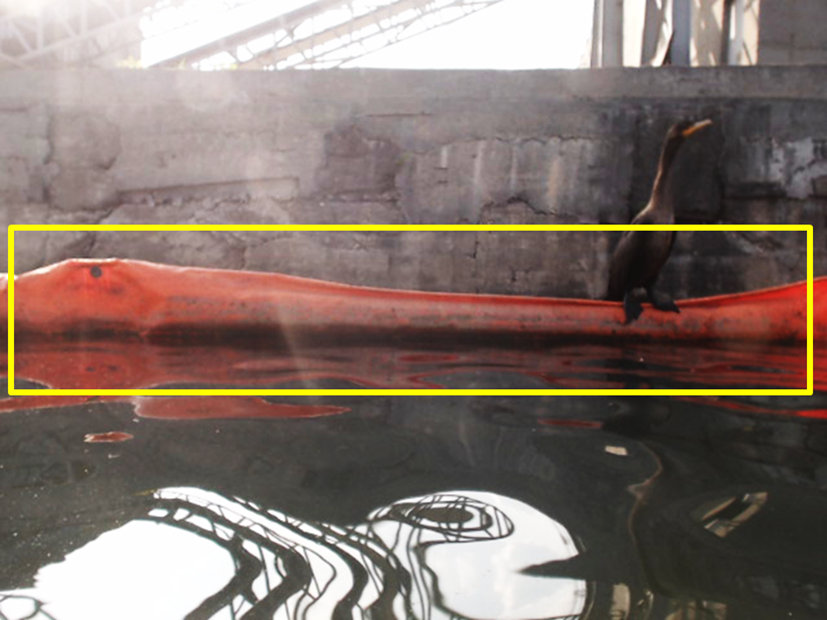

Supplement: S1 File — (ZIP) [file pone.0211907.s002.zip › Image17.png]

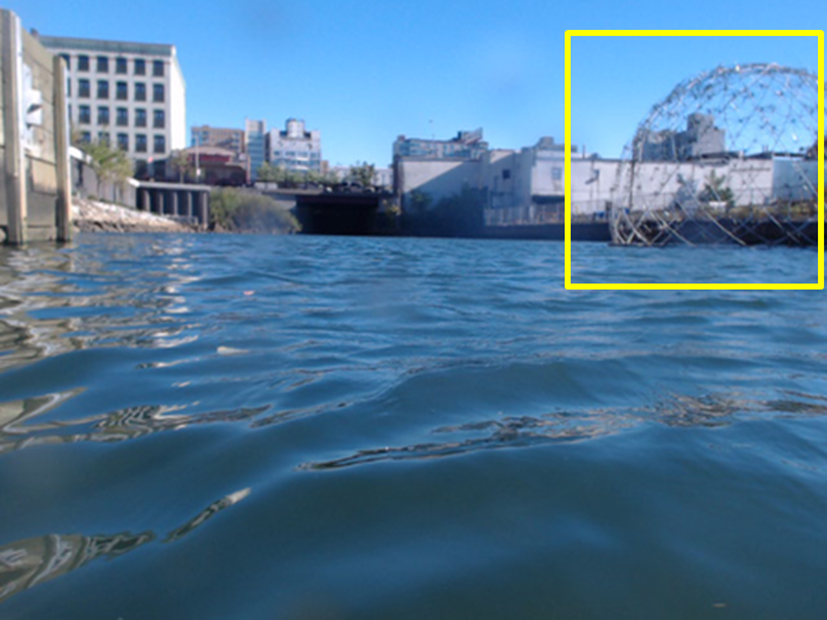

Supplement: S1 File — (ZIP) [file pone.0211907.s002.zip › Image16.png]

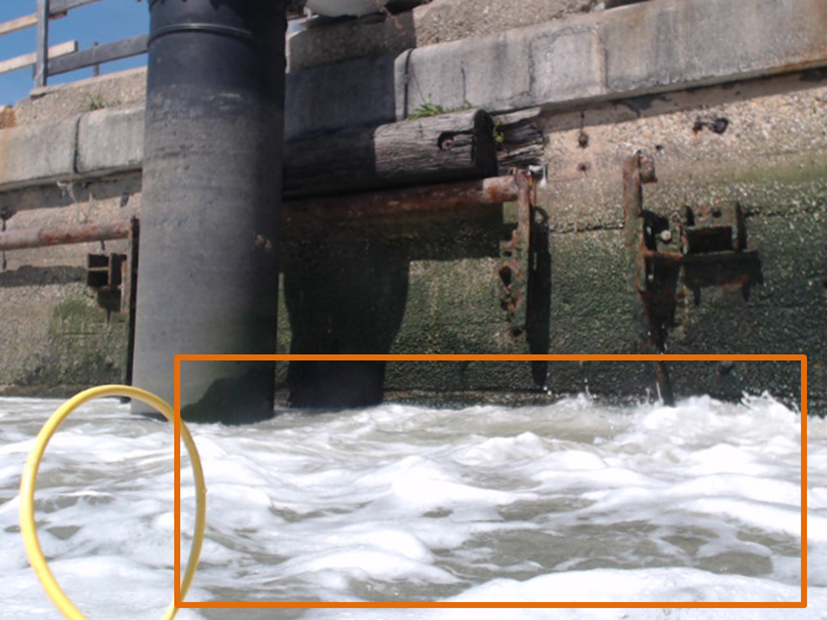

Supplement: S1 File — (ZIP) [file pone.0211907.s002.zip › Image12.png]

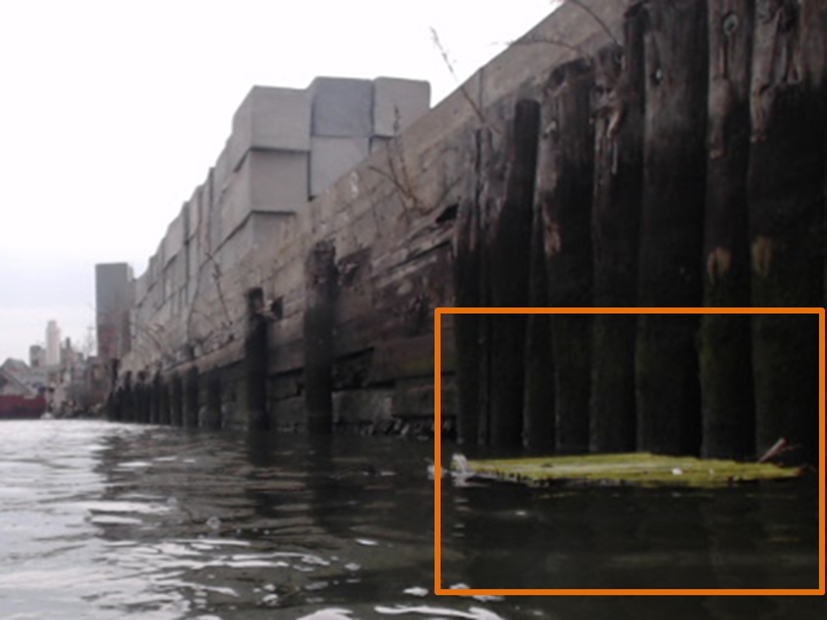

Supplement: S1 File — (ZIP) [file pone.0211907.s002.zip › Image13.png]

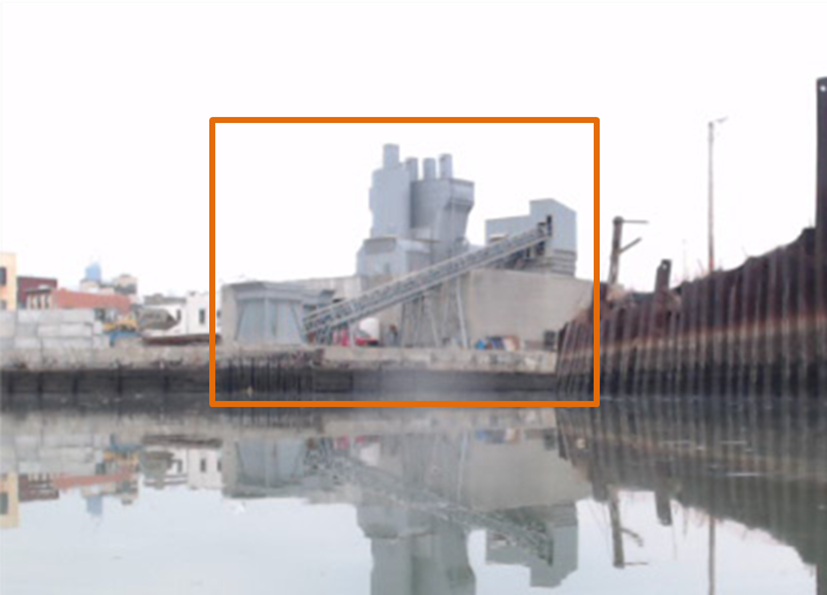

Supplement: S1 File — (ZIP) [file pone.0211907.s002.zip › Image11.png]

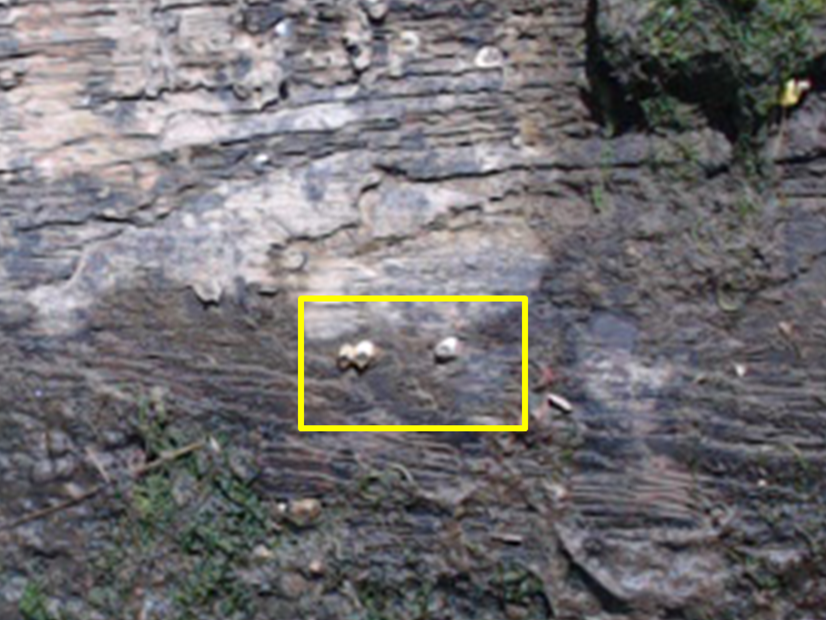

Supplement: S1 File — (ZIP) [file pone.0211907.s002.zip › Image10.png]

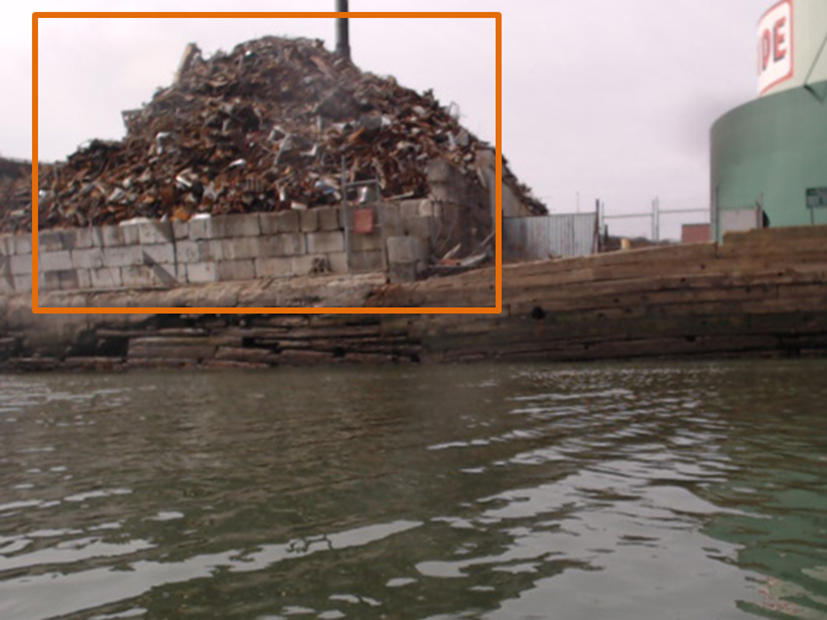

Supplement: S1 File — (ZIP) [file pone.0211907.s002.zip › Image21.png]

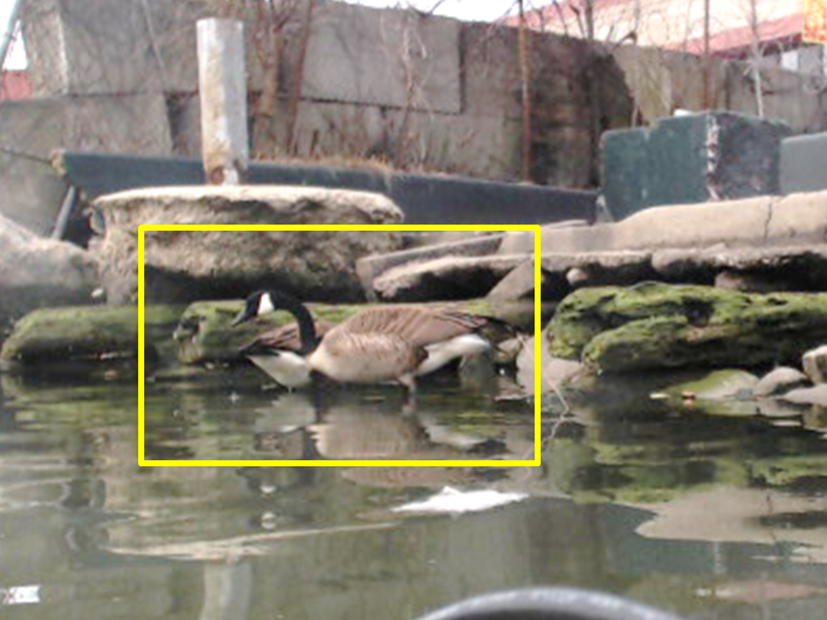

Supplement: S1 File — (ZIP) [file pone.0211907.s002.zip › Image20.png]

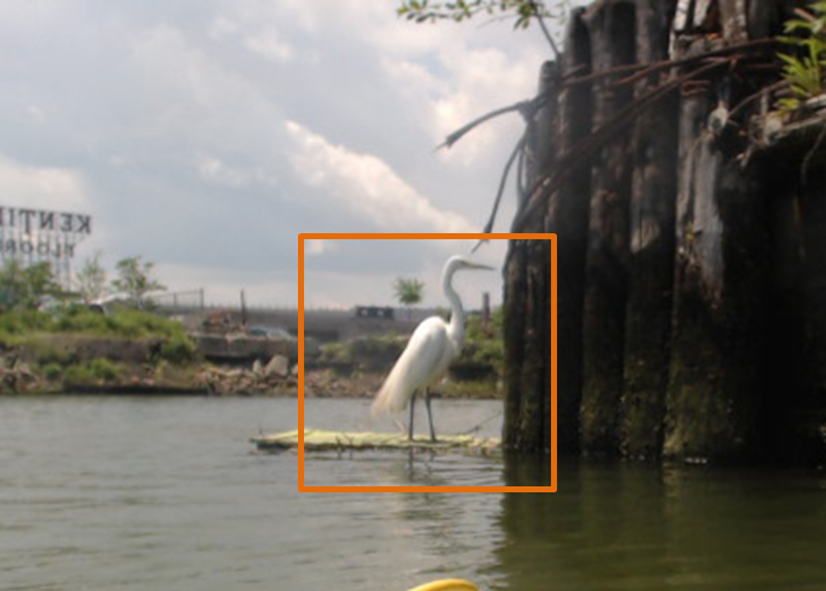

Supplement: S1 File — (ZIP) [file pone.0211907.s002.zip › Image22.png]

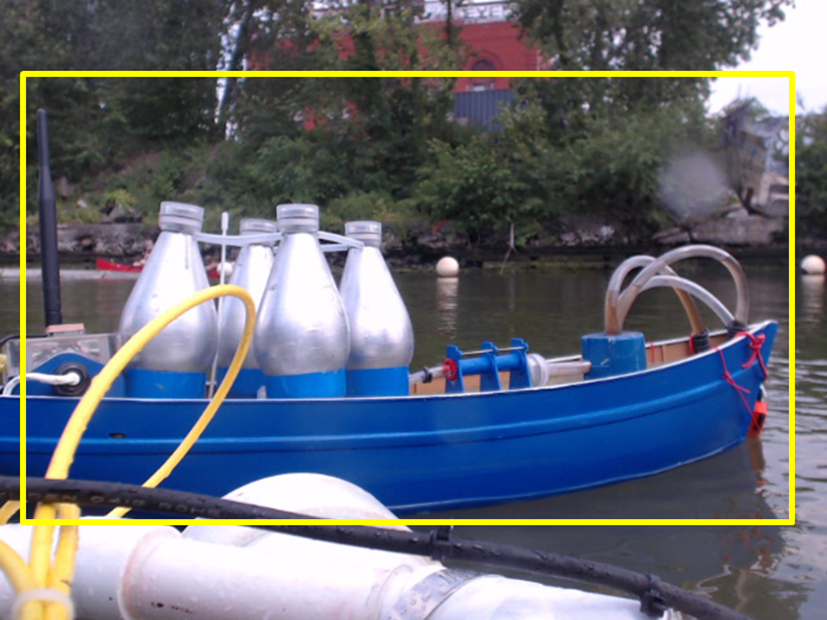

Supplement: S1 File — (ZIP) [file pone.0211907.s002.zip › Image23.png]

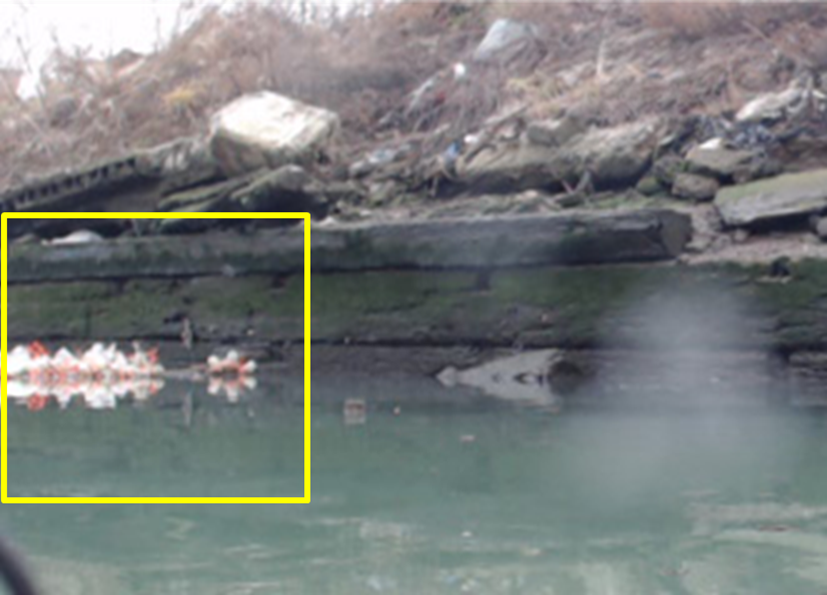

Supplement: S1 File — (ZIP) [file pone.0211907.s002.zip › Image27.png]

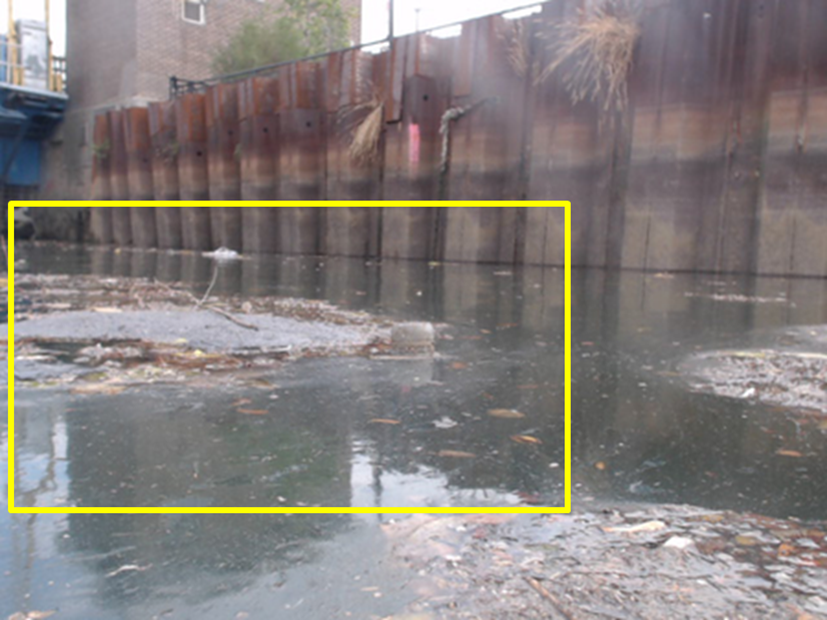

Supplement: S1 File — (ZIP) [file pone.0211907.s002.zip › Image26.png]

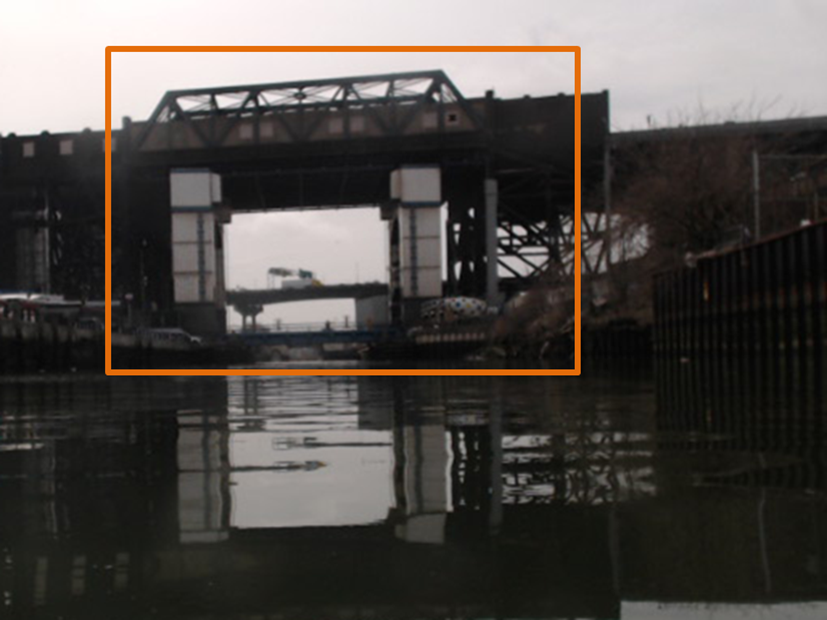

Supplement: S1 File — (ZIP) [file pone.0211907.s002.zip › Image18.png]

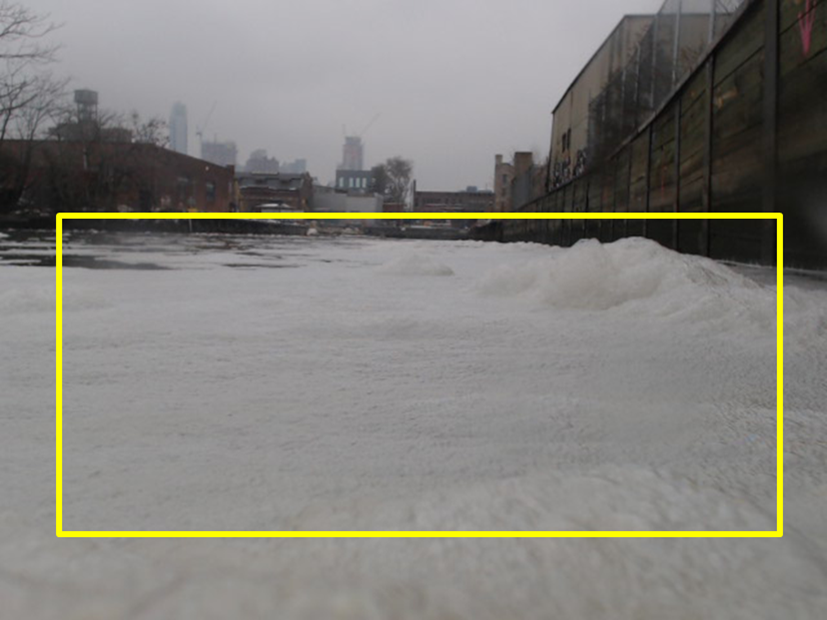

Supplement: S1 File — (ZIP) [file pone.0211907.s002.zip › Image30.png]

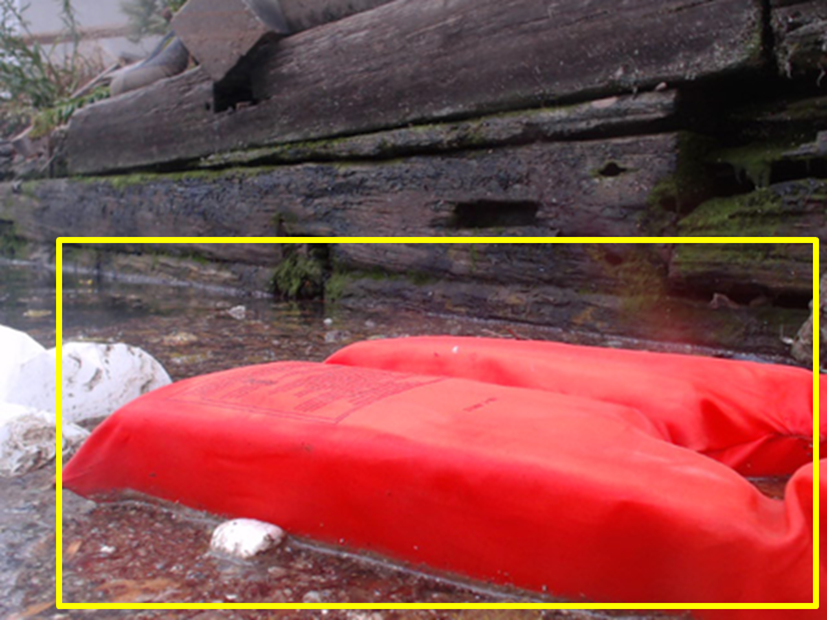

Supplement: S1 File — (ZIP) [file pone.0211907.s002.zip › Image24.png]

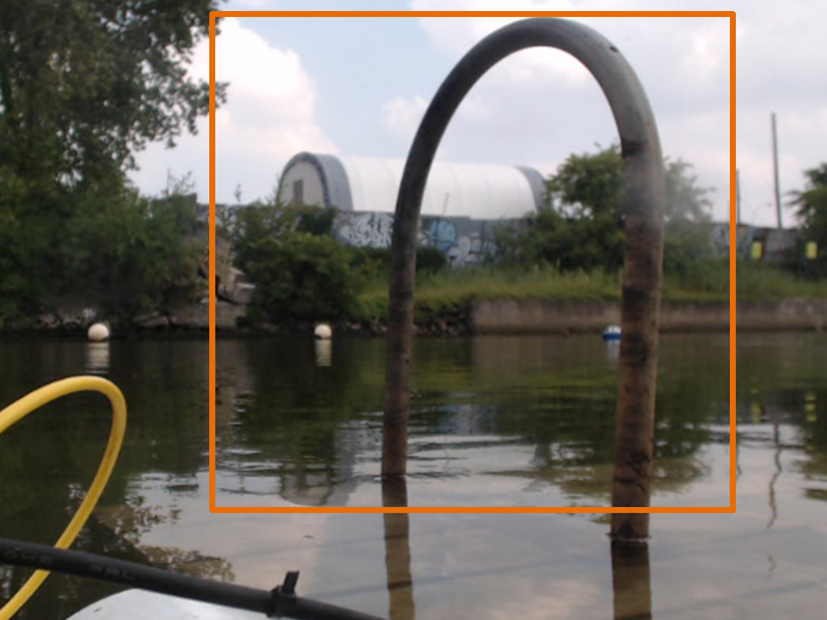

Supplement: S1 File — (ZIP) [file pone.0211907.s002.zip › Image25.png]

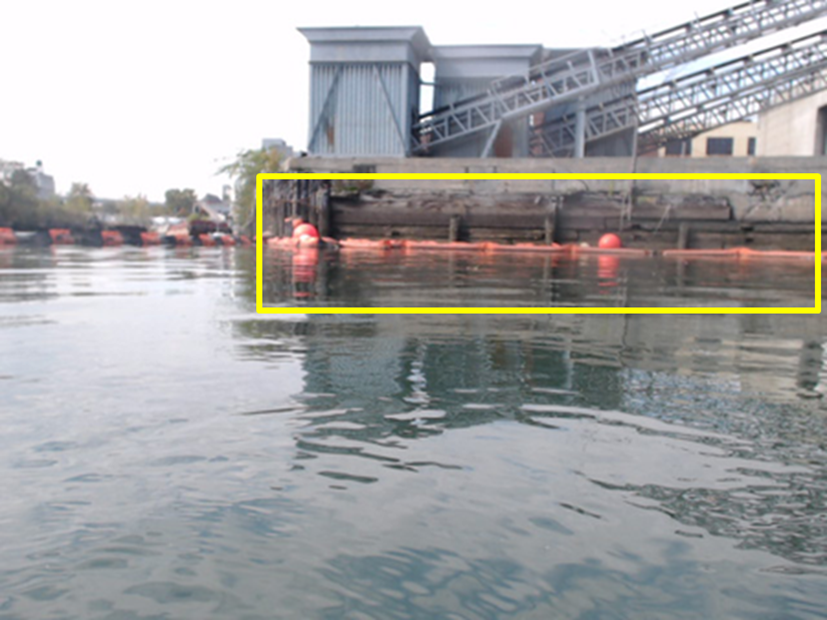

Supplement: S1 File — (ZIP) [file pone.0211907.s002.zip › Image31.png]

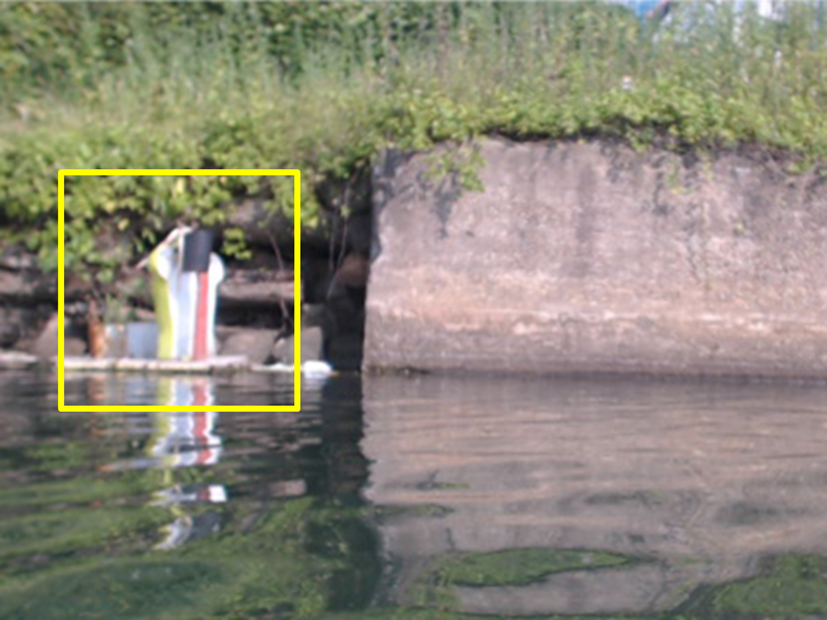

Supplement: S1 File — (ZIP) [file pone.0211907.s002.zip › Image19.png]

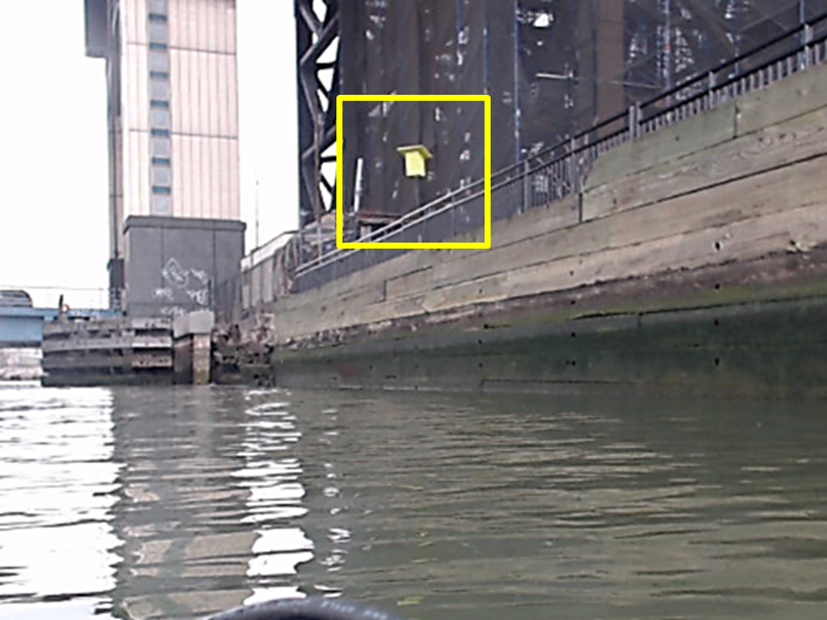

Supplement: S1 File — (ZIP) [file pone.0211907.s002.zip › Image1.png]

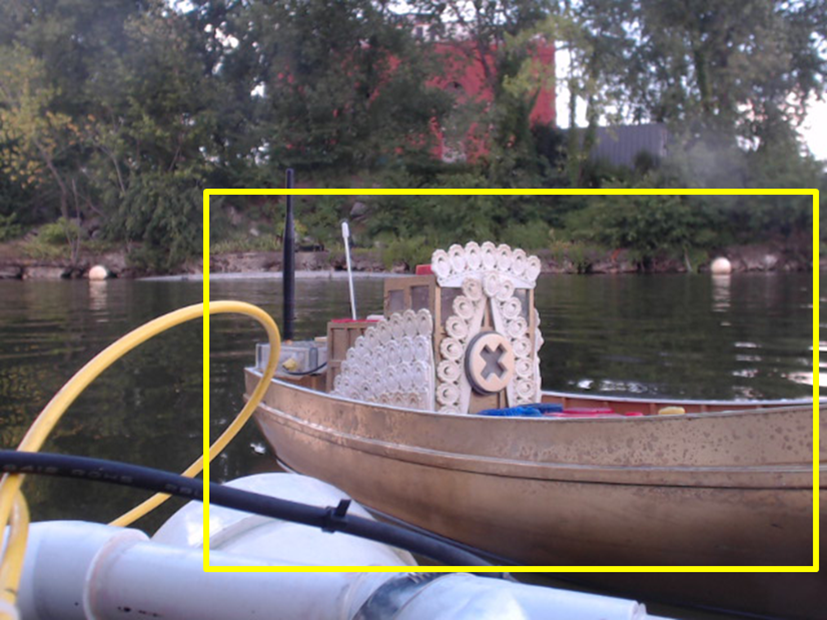

Supplement: S1 File — (ZIP) [file pone.0211907.s002.zip › Image2.png]
